# Epithelial-mesenchymal transition in colorectal cancer metastasis and progression: molecular mechanisms and therapeutic strategies

**DOI:** 10.1038/s41420-025-02593-8

**Published:** 2025-07-22

**Authors:** Fangfang Nie, Xue Sun, Jizhuo Sun, Jingdong Zhang, Yuanhe Wang

**Affiliations:** 1 Liaoning University of Traditional Chinese Medicine, Huanggu District, Shenyang, Liaoning China; 2 Medical Oncology Department of Gastrointestinal Cancer, Cancer Hospital of China Medical University, Cancer Hospital of Dalian University of Technology, Liaoning Cancer Hospital & Institute, Shenyang, Liaoning China

**Keywords:** Gastrointestinal cancer, Epithelial-mesenchymal transition

## Abstract

Colorectal cancer (CRC) continues to be a major contributor to cancer-associated death, with metastatic disease posing substantial therapeutic challenges. The epithelial-mesenchymal transition (EMT) orchestrates the transformation of polarized epithelial cells into motile mesenchymal phenotypes, characterized by enhanced migratory capacity and invasive properties. EMT is central to CRC metastasis and progression, particularly concerning its contribution to invasion, internal infiltration, and colonization. Beyond metastasis, EMT facilitates cancer cells’ adaptation to diverse microenvironments, gain of stem cell-like characteristics, metabolic reprogramming, and evasion of therapeutic interventions. EMT signatures are emerging as potential prognostic biomarkers, offering valuable insights for real-time disease surveillance and personalized therapeutic strategies. Targeting EMT presents a promising therapeutic avenue to improve drug sensitivity and counteract resistance in CRC. This review systematically examines the molecular mechanisms regulating EMT in CRC, including key transcription factors; post-translational and epigenetic modifications; non-coding RNAs; and pivotal signaling pathways. Additionally, we evaluate the clinical implications of EMT in CRC progression and metastasis and critically assess emerging therapeutic strategies targeting EMT. This study lays the groundwork for developing more efficient interventions to mitigate metastasis and enhance treatment outcomes and patient survival by elucidating the intricate molecular networks that govern EMT and its contributions to CRC pathology.

## Facts

- EMT drives CRC progression, metastasis, and therapy resistance by converting epithelial cells to motile mesenchymal forms.
- EMT is regulated by a complex network of transcription factors (e.g., SNAIL, ZEB, TWIST), post-translational modifications, epigenetic changes, non-coding RNAs, and signaling pathways (e.g., Wnt/β-catenin, TGF-β, PI3K/AKT).
- EMT is intricately linked to the acquisition of cancer stem cell-like properties, interactions with the tumor microenvironment (TME), metabolic reprogramming, and the development of drug resistance in CRC.
- Targeting EMT directly or indirectly through its regulatory pathways is a promising therapeutic strategy to improve drug sensitivity, counteract resistance, and mitigate metastasis in CRC.

## Questions

- How do partial/hybrid EMT states specifically drive CRC metastasis and therapy resistance, and can they be effectively targeted?
- Which reliable dynamic biomarkers can monitor EMT status in real-time to guide personalized CRC treatment and predict outcomes?
- When can inducing MET therapeutically inhibit CRC metastatic colonization without promoting the spread of already disseminated cells?

## Introduction

Colorectal cancer (CRC) remains one of the most prevalent occurring malignancies globally and a principal contributor to cancer-associated deaths [1]. Despite advances in early detection and treatment, metastatic CRC presents formidable therapeutic challenges, with a 5-year survival rate ~14% for distant metastases, highlighting an urgent need for innovative interventions [2].

Epithelial-mesenchymal transition (EMT), a dynamic cellular reprogramming process fundamental to embryogenesis and tissue regeneration, promotes tumor dissemination and metastasis when aberrantly activated in cancer [3]. The inherent plasticity of EMT allows tumor cells to adapt to varying microenvironmental challenges during metastasis [4]. The heterogeneity of EMT expression patterns across distinct cancer categories underscores its complex role in tumor biology [5], necessitating a comprehensive understanding of its effectors to delineate its precise contributions.

In CRC, EMT has emerged as a central mechanism driving progression and metastasis [6, 7]. Evidence indicates EMT is intricately connected to other tumor hallmarks, including stem cell-like properties, metabolic reprogramming, and alterations in the TME [8–10]. These interconnected mechanisms synergistically regulate CRC progression and metastatic potential, presenting both challenges and opportunities for therapeutic intervention. Notably, EMT signatures show promise as prognostic biomarkers, particularly concerning circulating tumor cells and metastatic disease [11]. EMT is also a significant factor in therapeutic resistance in CRC, often leading to treatment failure [12, 13]. Thus, targeting EMT represents a promising approach to counteract treatment resistance and optimize patient prognosis. This review systematically dissects the molecular mechanisms of EMT in CRC, evaluates its clinical implications in disease progression and metastasis, and critically assesses emerging EMT-targeted therapies, aiming to inform innovative diagnostic and therapeutic strategies.

## Fundamental aspects of EMTEMT

**Fig. 1:**
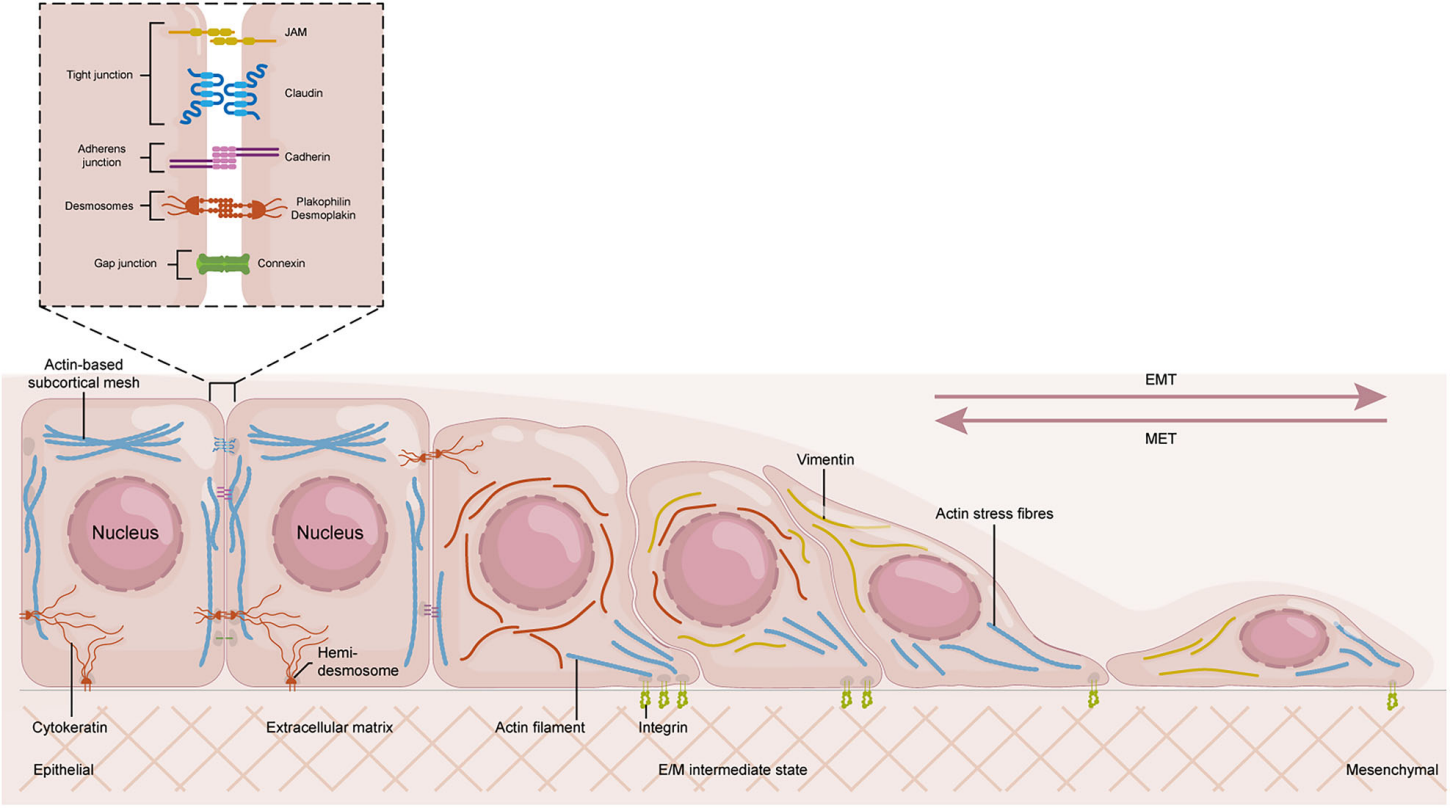
From epithelial to mesenchymal. Cellular Transformation and Plasticity in EMT. Epithelial cells exhibit apical–basal polarity with cell–cell and cell–matrix attachments. Intercellular junctions provide adhesion and communication, maintaining tissue stability and integrity. In contrast, mesenchymal cells lack functional epithelial junctions and exhibit back–front polarity. These cells contain vimentin-based intermediate filaments and use integrin-containing focal adhesions to attach to the extracellular matrix. As intercellular junctions dissolve and cell polarity is lost, cytoskeletal rearrangements transform epithelial cells into mesenchymal cells, enhancing motility and invasiveness. The accumulated loss or gain of epithelial/mesenchymal (E/M) characteristics moves cells into various intermediate states in a reversible manner. These states demonstrate remarkable plasticity, allowing cells to differentiate into various types and migrate collectively.

EMT is a dynamic, reversible cellular transformation during which epithelial cells experience significant biochemical and morphological changes to become mesenchymal [14] (Fig. 1). Epithelial tissues exhibit a highly organized architecture, underpinned by specialized surface proteins and a robust cytoskeleton that maintains tissue integrity and restricts cell migration [15]. Surface proteins facilitate the formation of intercellular junctions, comprising tight junctions, adherens junctions, desmosomes, and gap junctions, which form cohesive layers by mediating cell–cell adhesion, regulating paracellular permeability, establishing apical–basal polarity, and facilitating metabolic coupling. The actin cytoskeleton and keratin-based intermediate filaments provide intrinsic mechanical stability, while hemidesmosomes anchor epithelial cells to the basement membrane via integrin-extracellular matrix (ECM) interactions [3]. In contrast, mesenchymal cells are typically elongated or spindle-shaped, exhibit loose cell–cell contacts, and possess a more flexible cytoskeleton rich in vimentin intermediate filaments. This structure, along with dynamic actin stress fibers, enables efficient tissue migration [16].

Key molecular and cellular hallmarks define the EMT progression. Among these is the downregulation of epithelial markers, particularly E-cadherin (encoded by the CDH1 gene), a principal component of adherens junctions essential for maintaining epithelial integrity. Other epithelial proteins, including claudins, cytokeratins, and occludins, are also commonly reduced. Concurrently, there is a significant upregulation or de novo expression of mesenchymal markers. These include N-cadherin (often involved in a “cadherin switch” with E-cadherin), vimentin (an intermediate filament protein characteristic of mesenchymal cells), and fibronectin (an ECM protein) [17]. This is accompanied by the proteolytic breakdown of the underlying basement membrane, a dense ECM layer that normally acts as a physical barrier to cell movement [14]. These molecular shifts disrupt cell junctions and reorganize the cytoskeleton, transforming cells from a static, cohesive epithelial state to a motile, invasive mesenchymal phenotype, thereby enhancing cancer cell motility and metastatic potential.

EMT is not a rigid, unidirectional process. Its inherent reversibility is highlighted by the mesenchymal-epithelial transition (MET), crucial in development and metastatic colonization. In cancer, EMT rarely manifests as a complete switch; instead, cells often undergo partial EMT, inducing hybrid epithelial/mesenchymal (E/M) states. These cells co-express both epithelial and mesenchymal markers, existing in a flexible phenotypic window that may confer enhanced metastatic potential by integrating collective epithelial migration with mesenchymal invasiveness [18]. The term “epithelial-mesenchymal plasticity” (EMP) more precisely captures this capability to exhibit a spectrum of E/M characteristics and dynamically transition between them [3]. This remarkable plasticity fosters intratumoral heterogeneity and endows tumor cells with the adaptability to respond to fluctuating microenvironmental signals and selective pressures encountered during progression and therapy.

## Regulatory mechanisms of EMT in CRCCRC

### Transcriptional regulation of EMT

**Fig. 2:**
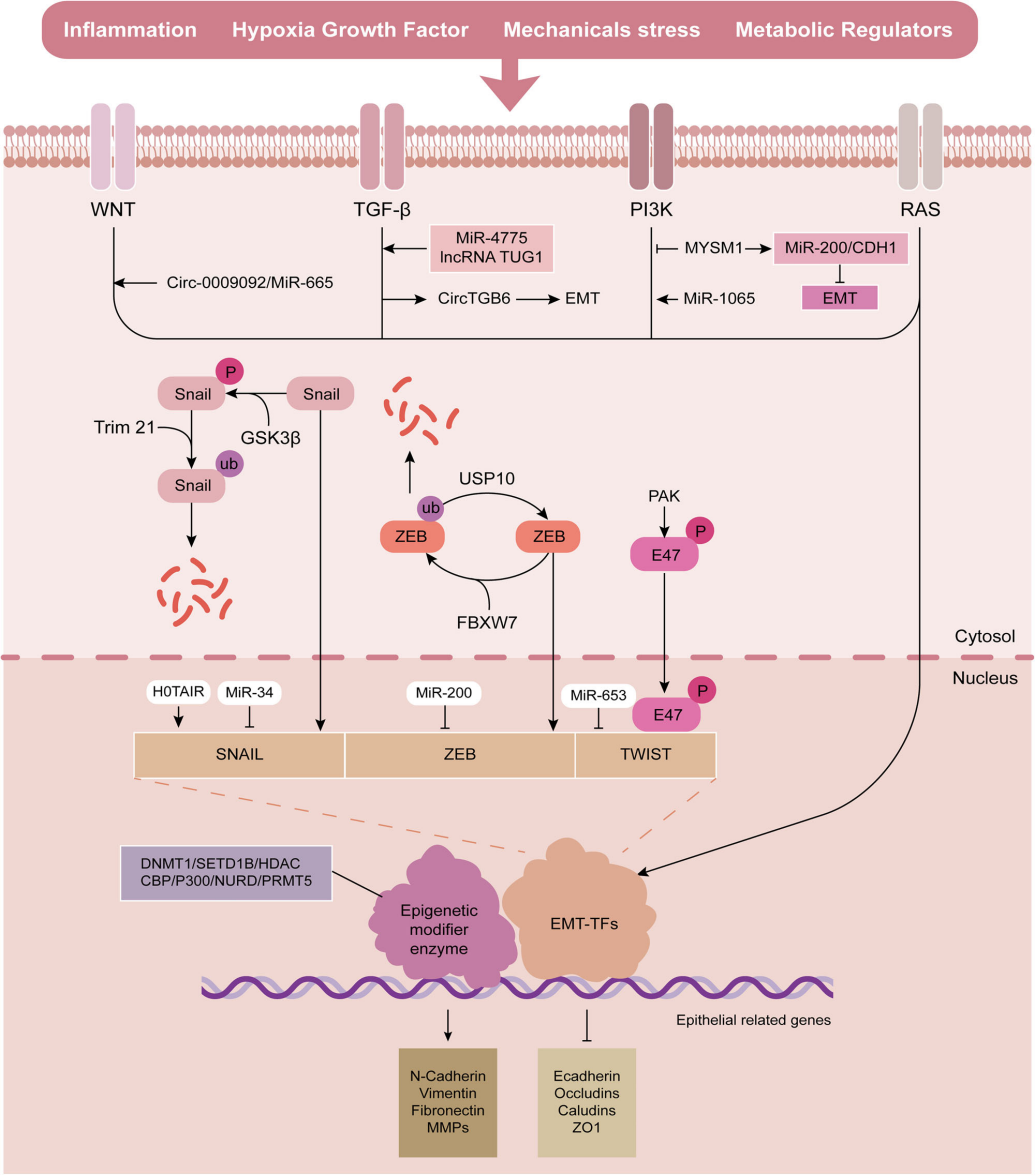
The EMT regulatory network in colorectal cancer. EMT is regulated by a complex network involving transcriptional regulation, post-translational modifications, epigenetic modifications, non-coding RNAs, and signaling pathways. These interconnected mechanisms collectively orchestrate the epithelial-mesenchymal transition in colorectal cancer progression.

The EMT-transcription factors (EMT-TFs) are of pivotal significance in orchestrating the EMT procedure, with key members including the SNAIL, ZEB, and TWIST families [19] (Fig. 2). These EMT-TFs drive the acquisition of EMT-associated cellular features by repressing epithelial phenotype-related genes while simultaneously inducing mesenchymal-related genes [20, 21]. Although significant functional redundancy exists among EMT-TFs, they operate within a dynamic and interdependent regulatory network, with their context-dependent expression patterns leading to unique, non-redundant contributions.

The SNAIL family members, particularly SNAIL1 (Snail) and SNAIL2 (Slug), are zinc finger (ZF) transcription factors. Both proteins contain ZF motifs that enable them to bind to E-box sequences in the promoter regions of target genes, thus repressing transcription and disrupting cell–cell adhesion [22, 23]. Slug predominantly depends on ZF3 and ZF4, while Snail’s functionality is primarily driven by ZF1 and ZF2, suggesting distinct DNA-binding specificities and regulatory targets [24]. Snail also engages in reciprocal interactions with Zeb1 UTR as competing endogenous RNAs (ceRNAs), promoting CRC progression [25]. In CRC, elevated overexpression of Slug and Snail is frequently correlated with increased invasiveness, metastatic potential, and dismal patient prognosis [26, 27].

The Zinc finger E-box-binding homeobox (ZEB) family, comprising ZEB1 and ZEB2, are also crucial transcriptional repressors of E-cadherin, binding to E-box elements in the CDH1 promoter and often recruiting co-repressors like C-terminal binding protein [28]. While ZEB1 primarily acts as a repressor, its interaction with p300 can confer activating potential through chromatin remodeling, enabling mesenchymal gene activation [29]. ZEB2, in collaboration with TWIST1, synergistically represses E-cadherin transcription [30]. Clinical research has demonstrated that high ZEB level in CRC are significantly links to reduced overall and disease-free survival (OS and DFS) [31].

TWIST1 and TWIST2, basic helix-loop-helix transcription factors, regulate EMT by heterodimerizing with E-proteins, enabling DNA binding and transcriptional repression of epithelial genes like CDH1 [32]. Non-acetylated TWIST1 recruits the NuRD complex to repress epithelial genes, whereas diacetylated TWIST1 interacts with BRD8 to activate mesenchymal gene loci and MYC expression [33]. TWIST1 is crucially involved in CRC progression, inducing chromosomal instability within the context of EMT, which enhances cellular heterogeneity and drives tumor advancement [34]. High TWIST1 expression in CRC has been linked to lymph node metastasis, reduced OS, and lower DFS [35]. Other TFs like SRY-box transcription factor 9 (SOX9) and Forkhead box (FOX) proteins (e.g., FOXS1, FOXF1, FOXM1) further enhance the regulatory complexity of EMT in CRC [36–40].

### Post-translational modifications

Post-translational modifications (PTMs) are pivotal in regulating EMT in CRC by fine-tuning the activity, stability, and subcellular localization of key EMT-related proteins (Fig. 2). Many EMT-TFs, including Snail and Twist, are intrinsically labile, swiftly degraded via the ubiquitin-proteasome system, with their cellular concentrations maintained through a strict balance between synthesis and degradation. Ubiquitination plays a central role. The E3 ubiquitin ligase FBXW7 promotes ZEB2 degradation, and its loss leads to EMT [13]. Conversely, ubiquitin-specific protease 10 (USP10) facilitates EMT by deubiquitinating and stabilizing ZEB1, preventing its proteasomal degradation [41]. Similarly, EDAR-associated death domain stabilizes Snail1 by engaging with the E3 ubiquitin ligase Trim21, preventing its ubiquitination [42]. Protein phosphorylation, another prevalent PTM, has a dual role. For instance, P21-activated kinase 5 (PAK5) phosphorylates E47, enhancing its nuclear localization and repression of E-cadherin, thereby promoting EMT [43]. However, kinases like glycogen synthase kinase-3β (GSK-3β) typically phosphorylate Snail1, Snail2, and Twist, priming them for recognition by E3 ligases and subsequent degradation [44].In addition to transcription proteins, phosphorylation also stabilizes cytoskeletal proteins, affecting EMT in CRC. HUNK directly phosphorylates GEF-H1, activating RhoA signaling to stabilize F-actin dynamics, counteracting EMT-driven invasion [45]. SUMOylation, another essential PTM, SUMOylation of the enhancer of EZH2 elevates its levels, and inhibiting this process reduces EZH2 expression while upregulating anti-metastatic genes like E-cadherin [46]. The rapid degradation of these TFs may prevent sustained EMT activation by transient microenvironmental signals, suggesting that targeting EMT-TF degradation could offer precise therapeutic benefits compared to broader signaling pathway inhibition.

### Epigenetic regulation

Pivotal epigenetic alterations, including DNA methylation and histone modifications, alongside their regulatory enzymes, have emerged as key modulators of the EMT program in CRC [47]. EMT-TFs including SNAIL, ZEB, and TWIST often act as docking platforms, recruiting various epigenetic modifiers to specific genomic loci to repress epithelial gene expression [48, 49] (Fig. 2). DNA hypermethylation in the promoter region of the CDH1 gene, encoding E-cadherin, is a common mechanism for its transcriptional silencing. Downregulation of DNA methyltransferase 1 (DNMT1) expression, for instance, has been demonstrated to inhibit EMT, migration, and cell proliferation in CRC by presumably reducing such aberrant methylation or affecting other key gene targets [50]. In addition to DNA methylation, histone modifications are also critical regulators of EMT. ZEB1 interacts with the promoter of the histone methyltransferase SETD1B, creating a positive feedback circuit by facilitating the SETD1B-dependent activation of the H3K4me3 histone mark, which facilitates ZEB1 promoter activity [51]. Additionally, histone deacetylase (HDAC) inhibitors, such as CBUD-1001, attenuate the motility of CRC cells via suppression of the EMT signaling cascade [52]. Myocyte enhancer factor 2D enhances ZEB1 expression by promoting histone acetylation at the ZEB1 promoter, likely through the recruitment of the p300 acetyltransferase [53]. Similarly, Ajuba, in cooperation with CREB-binding protein and TWIST, forms a ternary complex at the Twist target promoters, increasing histone acetylation and activating transcription of mesenchymal genes like N-cadherin [54]. These epigenetic modifications often function synergistically. For example, ZEB2, interacting with TWIST1, protein arginine methyltransferase 5 (PRMT5), and the NuRD complex, forms a repressive multicomplex, inducing the epigenetic silencing of E-cadherin and promoting EMT and metastasis [30]. The reversible nature of these epigenetic changes, independent of DNA sequence alterations, underscores their significant role in defining the plasticity and adaptability of the EMT process.

### Non-coding RNA

Non-coding RNAs (ncRNAs) have emerged as critical modulators of the EMT process in CRC, establishing an elaborate regulatory network that significantly influences CRC progression, metastasis, and poor prognosis (Fig. 2).

MicroRNAs (miRNAs) are critical regulators of EMT. Several miRNA families act as central regulators of EMT in CRC. For example, the miR-200 family powerfully suppresses EMT by directly targeting ZEB1 and ZEB2 mRNA, key transcription factors repressing E-cadherin. This forms a well-characterized double-negative feedback loop enabling dynamic EMT regulation [55–59]. Similarly, the miR-34 family inhibits EMT by targeting SNAIL [60–62]. Their downregulation has been linked to metastasis and advanced-stage CRC. Other miRNAs, like miR-653-3p, can regulate the SIRT1/TWIST1 axis, promoting EMT and genomic instability [63]. Furthermore, some miRNAs target mesenchymal genes, such as miR-17-5p, which suppresses vimentin expression and inhibits EMT in CRC [64]. On the other hand, certain miRNAs can indirectly facilitate EMT. Metastatic CRC cells release exosomal miR-335-5p, which facilitates invasion via RASA1 downregulation, and exosomal miR-27b-3p promotes metastasis by modulating vascular permeability [65, 66].

Long non-coding RNAs (lncRNAs) govern gene expression via diverse mechanisms, including acting as miRNA sponges (ceRNAs), guiding chromatin-modifying enzymes to specific genomic loci, or modulating mRNA stability and translation. Many lncRNAs have emerged as key players in CRC EMT. For instance, HOTAIR promotes EMT and CRC progression by suppressing HNF4α through the recruitment of SNAIL, thus enhancing invasion and migration [67]. MALAT1 acts as a ceRNA by sequestering miRNAs, while LINC00543 inhibits XPO5-mediated nuclear-cytoplasmic transport of pre-miR-506-3p, thereby reducing mature miR-506-3p levels. This interaction enhances EMT and promotes tumorigenesis and metastasis in CRC [68, 69]. HDAC2 deficiency promotes metastasis and EMT via upregulation of lncRNA H19 [70]. LncRNA ATB targeting miR-200c inhibits apoptosis and promotes proliferation in CRC [71].

Circular RNAs (circRNAs), a novel ncRNA class, are gaining recognition in CRC. For instance, hsa_circ_0001666 functions as a ceRNA for miR-576-5p, preventing protocadherin-10 downregulation and thereby suppressing EMT [72]. Conversely, circXPO1 upregulation in CRC correlates with poor survival [73]. These ncRNAs add profound complexity and offer potential therapeutic targets in EMT regulation in CRC.

### Signaling pathways in CRC EMT

**Fig. 3:**
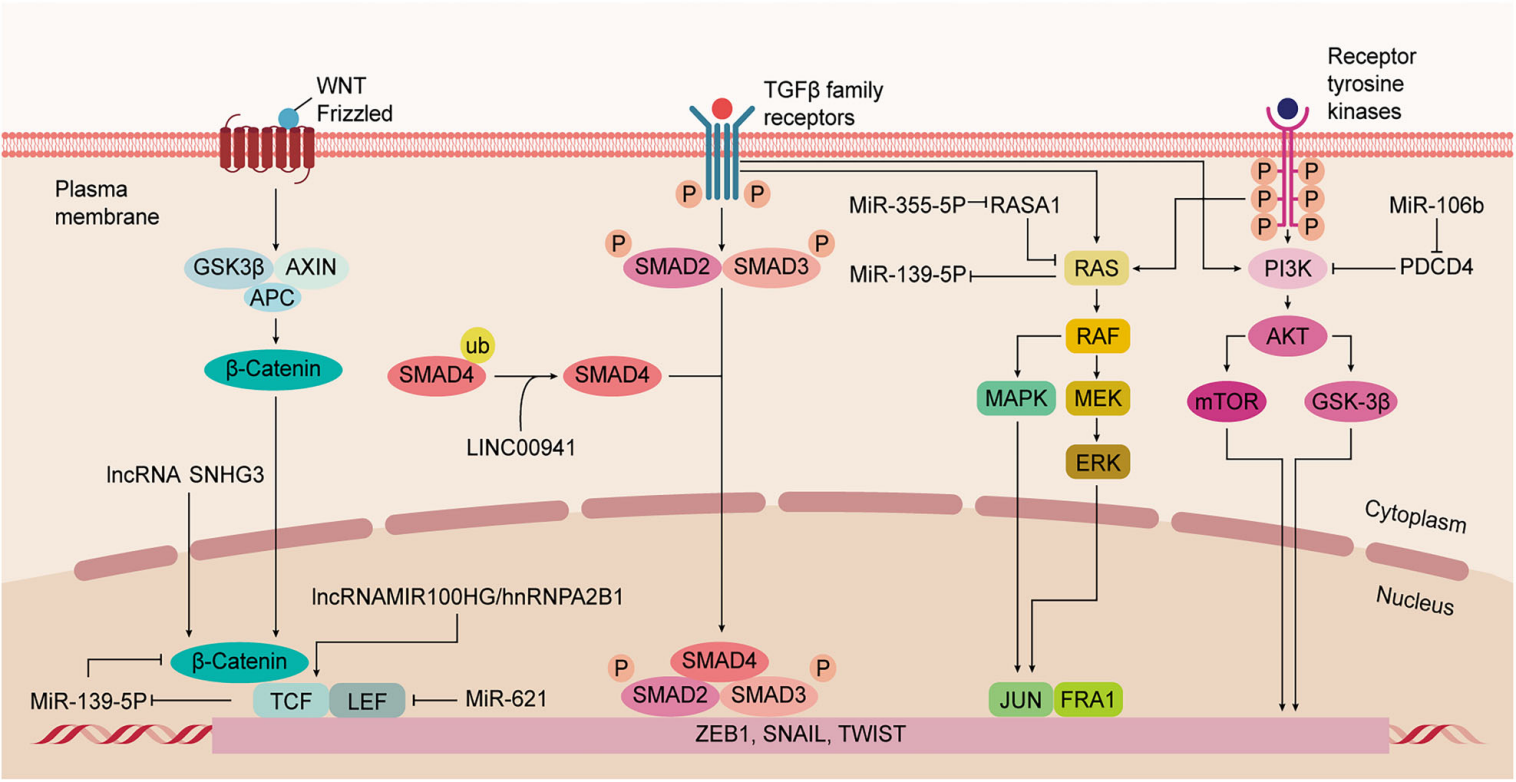
Signaling pathways regulating epithelial-mesenchymal transition in CRC. Multiple signaling pathways, including Wnt/β-catenin, TGF-β, and PI3K-AKT cascades, regulate EMT in colorectal cancer. These pathways modulate key transcription factors such as SNAIL, ZEB, and TWIST. Additionally, non-coding RNAs play crucial roles in fine-tuning this regulatory network, collectively driving EMT processes.

The Initiation of EMT-TFs and the subsequent EMT program in CRC are driven by a convergence of multiple, often interconnected, extracellular and intracellular signaling pathways. Moreover, ncRNAs fine-tune and amplify the regulatory effects of these signaling cascades. These pathways are intricately interconnected and engage in crosstalk, which is essential for EMT regulation (Fig. 3).

Aberrant Initiation of the Wnt/β-catenin is a hallmark of most CRCs, typically initiated by mutations in genes like APC or CTNNB1 (encoding β-catenin). Canonical Wnt signaling induces β-catenin stabilization and cytoplasmic accumulation. Stabilized β-catenin translocates to the nucleus, associating with TCF/LEF to activate target genes involved in proliferation, survival, and EMT [74]. Key EMT-TFs, like SNAIL1 and ZEB1, are significant downstream targets [75]. Additionally, FOXS1 also Orchestrates EMT through Wnt/β-catenin, contributing to CRC progression [36]. LEF1, a β-catenin cofactor, is crucial for SNAIL1-mediated invasion, and SNAIL1 can form complexes with β-catenin-LEF1 to further drive EMT [76]. Negative regulators like Axin2 also modulate this pathway; for instance, Axin2 inhibits GSK-3β-mediated SNAIL1 degradation, and suppressing Axin2 can promote E-cadherin expression and reduce EMT [77].

NcRNAs extensively regulate the Wnt/β-catenin pathway. For example, aberrant Wnt activation represses miR-139-5p via TCF4, creating a feedback circuit that enhances EMT in KRAS-mutant cells [78]. Conversely, miR-621 suppresses metastasis by directly targeting LEF1, demonstrating the potential for miRNAs in reversing EMT and metastasis [79]. LncRNA MIR100HG regulates TCF7L2 mRNA stability through interactions with hnRNPA2B1, forming a feedback loop that activates Wnt signaling, inducing EMT and contributing to cetuximab resistance [80]. Exosomal lncRNA SNHG3 promotes β-catenin expression by facilitating hnRNPC-mediated RNA stabilization, driving EMT and metastasis [81]. In addition, circRNA hsa_circ_0009092 inhibits EMT by sponging miR-665, thereby suppressing Wnt/β-catenin signaling [82].

TGF-β signaling initiates with ligand binding to TβRII, which activates TβRI kinase to phosphorylate SMAD2/3. Phosphorylated SMAD2/3 partners with SMAD4, forming a complex that translocates to the nucleus to orchestrate the expression of genes involved in EMT and metastasis [83]. In CRC progression, mutations activating the TGF-β pathway can induce SNAIL-mediated transcription of solute carrier family 14 member 1, which stabilizes TβRII, thereby amplifying TGF-β signaling and promoting EMT and liver metastasis [84]. Traditional Chinese Medicine formulations like Pien Tze Huang have been shown to inhibit TGF-β1 signaling, reducing the expression of key pathway components and downstream targets like ZEB1/2, thus reversing EMT [85].

SMAD7, an inhibitory SMAD, antagonizes TGF-β signaling by promoting TβRI degradation. MiR-4775 suppresses SMAD7 expression, elevating p-SMAD2/3 levels and activating downstream signaling [86]. LncRNAs also play significant roles in regulating TGF-β-mediated EMT. For instance, lncRNA TUG1 knockdown attenuates TGF-β-induced TWIST1 upregulation, inhibiting CRC cell invasion and reducing lung metastasis [87]. LINC00941 activates EMT by directly binding to the MH2 domain of SMAD4, stabilizing SMAD4 and enhancing TGF-β signaling [88]. CircRNAs, such as circITGB6, are also involved in TGF-β-induced EMT. CircITGB6 promotes EMT through podoplanin, and silencing circITGB6 suppresses liver metastasis in CRC, making it a potential therapeutic target [89]. Given TGF-β‘s potent EMT-inducing activity in advanced CRC, its pathway is a key therapeutic target.

The PI3K/AKT pathway drives EMT through growth factor-mediated RTK activation. Ligand-bound RTKs activate PI3K, which generates PIP3 to recruit and phosphorylate AKT. Activated AKT then targets downstream effectors (e.g., GSK3β, mTOR) to promote EMT-associated processes like invasion and stemness [90]. In CRC, the PI3K/AKT pathway influences EMT by regulating EMT-TFs. Extracellular matrix protein 1 promotes CRC metastasis and EMT by the PI3K/AKT/GSK3β/Snail axis [91]. Additionally, the overexpression of inosine 5’-monophosphate dehydrogenase type II facilitates the G1/S cell cycle transition and enhances cell EMT and invasion through the PI3K/AKT/mTOR axis [92]. Elevated miR-106b activates the PI3K/AKT/mTOR axis by post-transcriptionally suppressing programmed cell death 4, contributing to M2 macrophage polarization, promoting EMT-dependent migratory, invasive, and metastatic phenotypes in CRC cells [93]. Furthermore, the deubiquitinase MYSM1 erases repressive H2AK119ub1 marks of miR-200/CDH1 promoters, thereby maintaining epithelial integrity. MYSM1 deficiency unleashes PI3K/AKT signaling through miR-200 suppression, driving EMT and metastatic progression in CRC [94]. Methyltransferase-like 14, an m6A RNA methylation regulator, also plays a role; its knockdown increases SOX4 expression (an EMT inducer) through reduced m6A methylation of SOX4 mRNA, enhancing EMT via the PI3K/AKT pathway [95].

Crosstalk between pathways is also evident; for instance, the TRIP13 inhibitor DCZ0415 blocks the TRIP13/FGFR4/STAT3 axis, achieving dual inhibition of NF-κB/Wnt pathways to suppress EMT-driven metastasis [96]. In hypoxic CRC, Nur77 promotes invasiveness by suppressing p63-dependent Dicer transcription, leading to reduced let-7i-5p expression. This destabilizes p110α mRNA, resulting in AKT activation, which subsequently enhances β-catenin signaling and CRC progression [97].

## EMT and CRC metastasis and progression

**Fig. 4:**
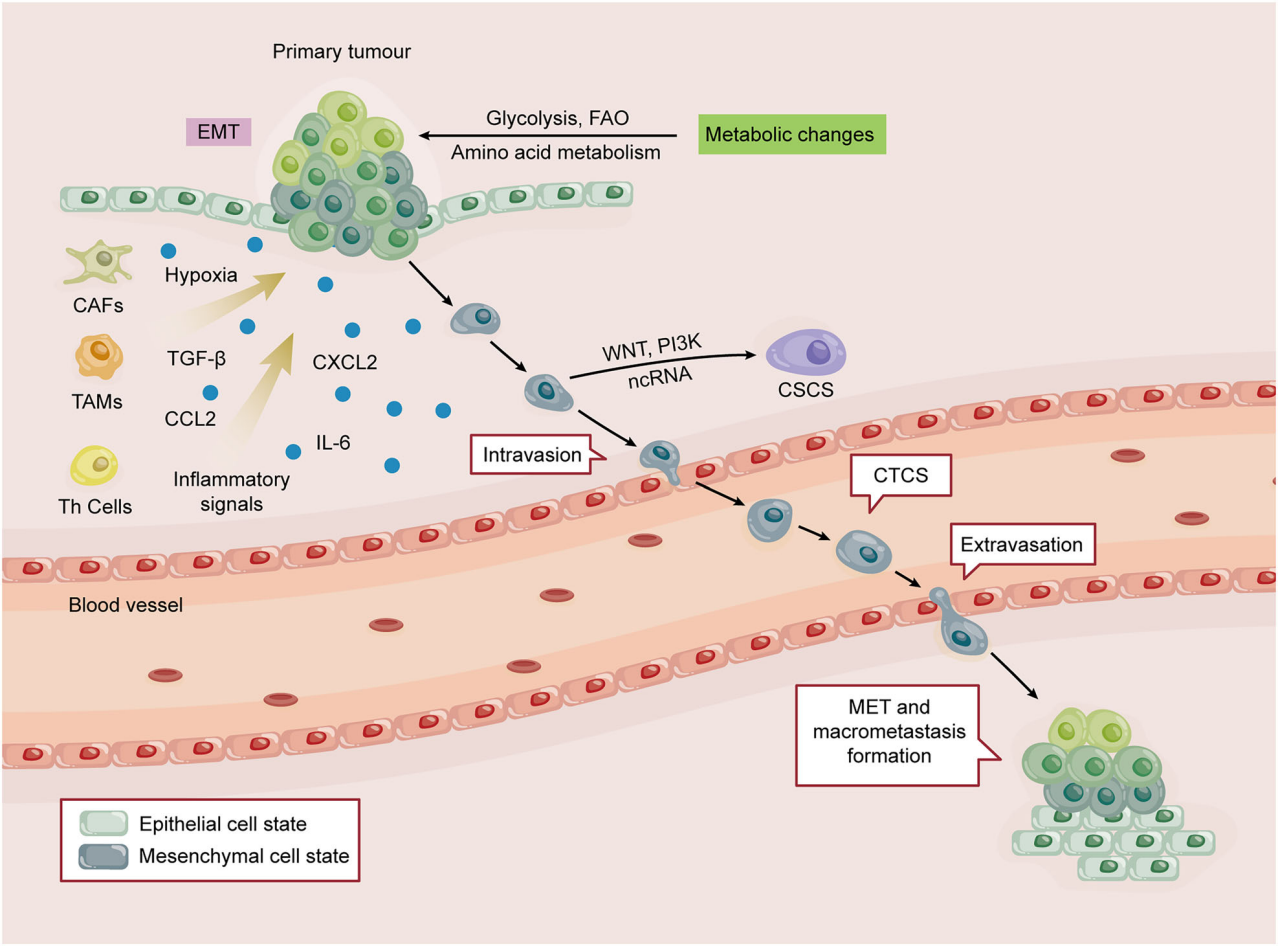
The role of EMT in the progression and metastasis of CRC. Colorectal cancer cells undergoing EMT progressively lose intercellular adhesions and acquire the capacity to degrade the basement membrane and ECM. This enables detachment from the primary tumor and invasion into surrounding tissues. EMT-driven cells enhance vascular permeability, facilitating intravasation into the bloodstream. Surviving circulating tumor cells withstand hemodynamic shear forces and resist anoikis before undergoing extravasation at distant sites, initiating metastatic colonization through MET. Within the tumor microenvironment, interactions between cancer cells and stromal components (immune cells, fibroblasts) generate EMT-inducing signals via cytokines and chemokines. Environmental stressors, such as hypoxia, inflammatory signals, and nutrient competition, drive metabolic adaptations like glycolysis, enhancing survival. EMT also confers stemness properties, enabling self-renewal and multi-lineage differentiation potential, ultimately promoting metastatic colonization.

The development of cancer metastasis is a complex, multi-phasic process known as the invasion-metastasis cascade, and EMT exerts a critical and intricate role throughout this cascade in CRC [98]. EMT influences every stage, from local invasion in the primary tumor to the eventual colonization of metastatic remote organs (Fig. 4).

Metastasis initiation in CRC typically begins with local invasion, heavily facilitated by EMT at the tumor’s invasive front. Here, CRC cells lose epithelial traits and gain mesenchymal features, reducing cell–cell adhesion and remodeling the cytoskeleton. These changes enable tumor cell dissociation from the primary mass, basement membrane penetration, and stromal invasion—critical steps for local dissemination and metastatic spread [99]. EMT further promotes local invasion by equipping cancer cells with the machinery to degrade the ECM. For instance, Gab2 overexpression can activate ERK1/2 signaling, inducing matrix metalloproteinases (MMPs) like MMP-7/9, which break down ECM components [100]. Post-invasion, EMT-driven motility enables CRC cells to penetrate the ECM and intravasate into blood/lymphatic vessels, initiating systemic dissemination, a step known as intravasation. Several mechanisms contribute to this step. EMT-induced CRC cells can weaken the vascular barrier, for example, by transferring exosomal miR-27b-3p to endothelial cells, thereby increasing vascular permeability [66]. Factors like thrombin-induced hypoxia-inducible factor-1α (HIF-1α) can upregulate TWIST, promoting cell motility critical for intravasation [101]. Fusobacterium nucleatum can also accelerate tumor progression by inducing neutrophil extracellular traps, promoting angiogenesis, and facilitating CRC cell migration [102]. The successful invasion of cancer cells leads to their presence in the bloodstream as Circulating Tumor Cells (CTCs). Once in the bloodstream, CTCs face a hostile environment characterized by anoikis (detachment-induced apoptosis), shear stress, and immune surveillance. EMT enhances the ability of CTCs to survive these challenges [103–105]. EMT-transformed cancer cells express diverse adhesion molecules that facilitate their attachment to the vascular endothelium of the target organ and extravasation [106]. For efficient colonization of a distant organ and formation of a clinically significant secondary tumor, MET is often necessary. While a mesenchymal phenotype is advantageous for invasion and dissemination, an epithelial phenotype is generally more conducive to proliferation and the formation of organized tumor structures [18]. Beyond these core steps, EMT is deeply intertwined with other critical aspects of CRC progression, including the gain of CSC-like characteristics, dynamic interactions with the TME, and metabolic reprogramming to fulfill the heightened energy requirements of metastatic cells [8–10].

## EMT and CRC stem cells

Cancer stem cells (CSCs) represent a critical tumor subpopulation characterized by self-renewal, tumor-initiating capacity, and long-term clonal expansion, driving tumor recurrence and metastatic colonization [5]. Emerging evidence indicates that CSC properties can be acquired through EMP programs, rather than being solely intrinsic [9] (Fig. 4). Notably, cells in a hybrid E/M state or undergoing partial EMT exhibit a greater propensity to gain stem cell-like characteristics compared to fully epithelial or mesenchymal cells, suggesting a “stemness window” between these differentiated states [5, 9].

The activation of EMT is intricately linked to the gain of stem cell characteristics, with key EMT-TFs playing pivotal roles in regulating stemness in CRC. For instance, type I collagen can activate the integrin α2β1/PI3K/AKT/Snail axis, enhancing both stemness and metastatic potential [107]. Therapeutic compounds like bufalin have been demonstrated to inhibit CRC tumorigenesis, EMT, and stemness by targeting axes such as C-Kit/Slug, which can form stemness-promoting feedback loops [108]. TM4SF1 has also emerged as a crucial mediator linking cancer stemness and EMT in CRC, potentially through the Wnt/β-catenin pathway and regulation of SOX2, a key factor in both processes [109]. Furthermore, dysregulated tight junction proteins like claudin-1 can promote EMT and metastasis by interacting with receptors such as ephrin type-A receptor 2, enhancing downstream AKT signaling and upregulating stemness markers like CD44 [110].

NcRNAs also significantly influence the maintenance and induction of EMT and stemness. Cancer-associated fibroblasts (CAFs) can bridge EMT and stemness by releasing miR-92a-3p-enriched exosomes, which drive tumor cell stemness and EMT [111]. LncRNAs like GATA2-AS1 can recruit proteins such as DDX3X to stabilize GATA2 mRNA, forming a self-reinforcing loop that promotes CRC cell proliferation, EMT, and stemness [112]. Similarly, SOX2-induced circRNAs, like circ_0026628, can sponge miRNAs (e.g., miR-346) and recruit proteins to activate pathways like Wnt/β-catenin, thereby promoting EMT and stemness [113]. These results underscore the intricate crosstalk among EMT, stemness, and ncRNAs, pointing to potential therapeutic strategies aimed at disrupting this network in CRC.

## EMT and TME in CRC

Emerging evidence highlights a reciprocal crosstalk between the TME and EMT in CRC. TME-derived signaling molecules drive tumor cell EMT, while EMT-activated cells reciprocally remodel TME components, fostering a pro-tumorigenic niche [114]. The TME orchestrates CRC progression by coordinating various cellular components, such as stromal and immune cells, and non-cellular signals like cytokines, chemokines, and hypoxia, which collectively induce EMT [115] (Fig. 4). For example, tumor-associated macrophages (TAMs) can drive CRC migration and invasion by activating the JAK2/STAT3/miR-506-3p/FoxQ1 signaling to induce EMT, simultaneously triggering CCL2-mediated macrophage recruitment in a feedforward loop [116]. TWEAK, secreted by T helper 17 (Th17) cells, binds to the Fn14 receptor on CRC cells, inducing EMT [117]. ZEB1 can regulate CAF-derived MYL9, which promotes CCL2 and TGF-β1 expression, altering the immune microenvironment and enhancing CRC progression [118]. Microenvironmental factors, including inflammatory signals and hypoxia, also activate EMT, highlighting the sophisticated interaction between tumor cells and their surrounding environment. For example, Fusobacterium nucleatum infection can induce neutrophil extracellular traps, promoting angiogenesis and facilitating EMT in CRC [102]. Hypoxia induces Nur77-mediated activation of the PI3K/Akt signaling, which further drives CRC EMT through suppression of Dicer/let-7i-5p [97].

Moreover, EMT significantly contributes to CRC immune evasion. It enhances the secretion of chemokines, recruits immunosuppressive cells, and upregulates immune checkpoint molecules, creating an immunosuppressive microenvironment that enables tumor cells to evade cytotoxic T lymphocyte (CTL) attacks [119]. For example, SNAIL-induced EMT promotes lung metastasis in CRC through CXCL2 secretion, attracting M2-type immunosuppressive macrophages and advancing tumor progression [26]. These dynamic interactions between EMT and the TME form a reciprocal cycle profoundly affecting CRC metastasis and progression.

## EMT and metabolic reprogramming in CRC

EMT is closely intertwined with metabolic reprogramming, as cancer cells must adapt their metabolism to fulfill the increased energy requirements associated with proliferation, motility, and survival during metastasis [10] (Fig. 4). A hallmark of this metabolic shift is the Warburg effect, where tumor cells favor glycolysis even in the presence of oxygen [120]. Pyruvate kinase M2 (PKM2), a key glycolytic enzyme, plays a role in this; nuclear PKM2 can partner with the TGF-β signaling cofactor TGIF2 to recruit HDAC3 to the E-cadherin promoter, suppressing its expression and facilitating EMT [121]. Targeting glycolysis, for instance with shikonin-loaded nanoparticles that inhibit cytoplasmic PKM2, can reduce tumor glycolysis and lactate flux. This not only suppresses EMT but can also remodel the TME by hindering myeloid-derived suppressor cell (MDSC) migration and activating dendritic cells [122].

Beyond glycolysis, alterations in fatty acid (FA) metabolism are significant. ACSL3 drives CRC EMT and metastasis by fueling fatty acid oxidation (FAO), generating ATP and NADPH to sustain redox homeostasis and power invasive behavior [123]. In CRC, acidosis-activated TGF-β2 can orchestrate partial EMT and FA metabolic reprogramming, promoting CD36 translocation to enhance FA uptake, which then fuels either triglyceride storage or ATP-generating oxidation [124]. Furthermore, glutaminolysis is implicated; compounds like curcumin can suppress CRC growth, metastasis, and EMT by inducing HIF-1α degradation and inhibiting glutaminase 1-driven glutaminolysis [125]. These metabolic adaptations are essential for sustaining the energetic and biosynthetic demands of cells undergoing EMT.

## Clinical therapeutic implications

### EMT as a prognostic marker

Given the pivotal role of EMT in CRC progression and metastasis, biomarkers related to this process are becoming vital for diagnosis, prognosis, and personalizing treatment [6, 7]. Clinical evidence supports multi-marker EMT analyses, often via immunohistochemistry, for tumor stratification [126]. In CRC, evaluating markers like vimentin, E-cadherin, claudin-1, and Snail-1 offers clinical value for identifying patients with lymph node metastases, advanced disease, or higher recurrence risk [127]. For instance, in CRC patients, cytoplasmic Snail expression and a novel EMT score independently predicted worse cancer-specific survival and correlated with adverse features like tumor budding and depleted memory T-cell infiltration, suggesting a tool to stratify high-risk patients for EMT-targeted adjuvant therapy regimens [128].

Liquid biopsies, particularly assessing EMT characteristics of CTCs, show considerable promise [129]. EMT-transformed CTCs can gain stem cell-like characteristics and promote immunosuppression, aiding their survival and metastatic potential. Thus, CTC heterogeneity due to EMT offers insights into metastatic risk [130]. A study in metastatic CRC patients demonstrated that a multi-marker CTC panel was significantly correlated with shorter PFS and OS, even identifying therapy-resistant patients earlier than conventional imaging [11]. This underscores the potential of EMT status in CTCs for real-time therapeutic monitoring and prognostic assessment in CRC.

### EMT and drug resistance

One of the most clinically challenging consequences of EMT is its profound contribution to therapy resistance in CRC [7]. This resistance is driven by multiple mechanisms, including inhibition of cell death, increased stemness, enhanced drug efflux, DNA damage repair, and metabolic alterations. For instance, the combined loss of miR-34a and miR-34b/c isoforms undermines p53-mediated control of proliferation, induces EMT, and diminishes chemosensitivity. This resistance is linked to stress-induced autophagy and upregulation of autophagy-associated genes following 5-fluorouracil (FU) exposure, which ultimately attenuates apoptosis [61]. Additionally, the SOX2-β-catenin/Beclin1/autophagy signaling orchestrates drug resistance, EMT and stemness in CRC, highlighting the intricate interplay among these mechanisms [131]. EMT-associated drug resistance is also mediated through the upregulation of ATP-binding cassette (ABC) transporters, which facilitate the efflux of chemotherapeutic agents from cancer cells. For example, the knockdown of TMEM97 reduces 5-FU resistance in CRC by modulating EMT and suppressing ABC transporter expression [132]. Furthermore, the ZEB2-driven EMT program activates the nucleotide excision repair (NER) pathway by upregulating the ERCC1 gene and other NER components. CRC cells overexpressing ERCC1 exhibit resistance to oxaliplatin in vivo [12]. TME plays a pivotal role in these dynamics; for instance, MDSCs secrete IL-23, which activates the STAT3-EMT pathway, inducing EMT and promoting chemoresistance in CRC [133]. Additionally, metabolic alterations, particularly those involving thiol oxidative stress, further influence EMT progression and drug sensitivity. Thiol metabolism regulates the expression and post-translational modifications of key molecules, such as transglutaminase 2, providing new avenues for targeting metabolic pathways to reverse EMT and overcome drug resistance [134].

### Targeted EMT for CRC

Given the critical role of EMT in CRC progression, metastasis, and resistance, targeting EMT represents a promising therapeutic strategy. Clinically, EMT-targeted therapy could be employed to overcome resistance to conventional treatments; for example, curcumin has been shown to mediate chemosensitization to 5-FU by miRNA-induced EMT suppression in chemoresistant CRC cells [135]. Additionally, based on EMT’s role in metastasis, such therapies could serve as adjuvant treatments to control metastatic spread by decreasing CSC proportions, preventing CTC colonization, and reducing their ability to form secondary tumors [136].

**Fig. 5:**
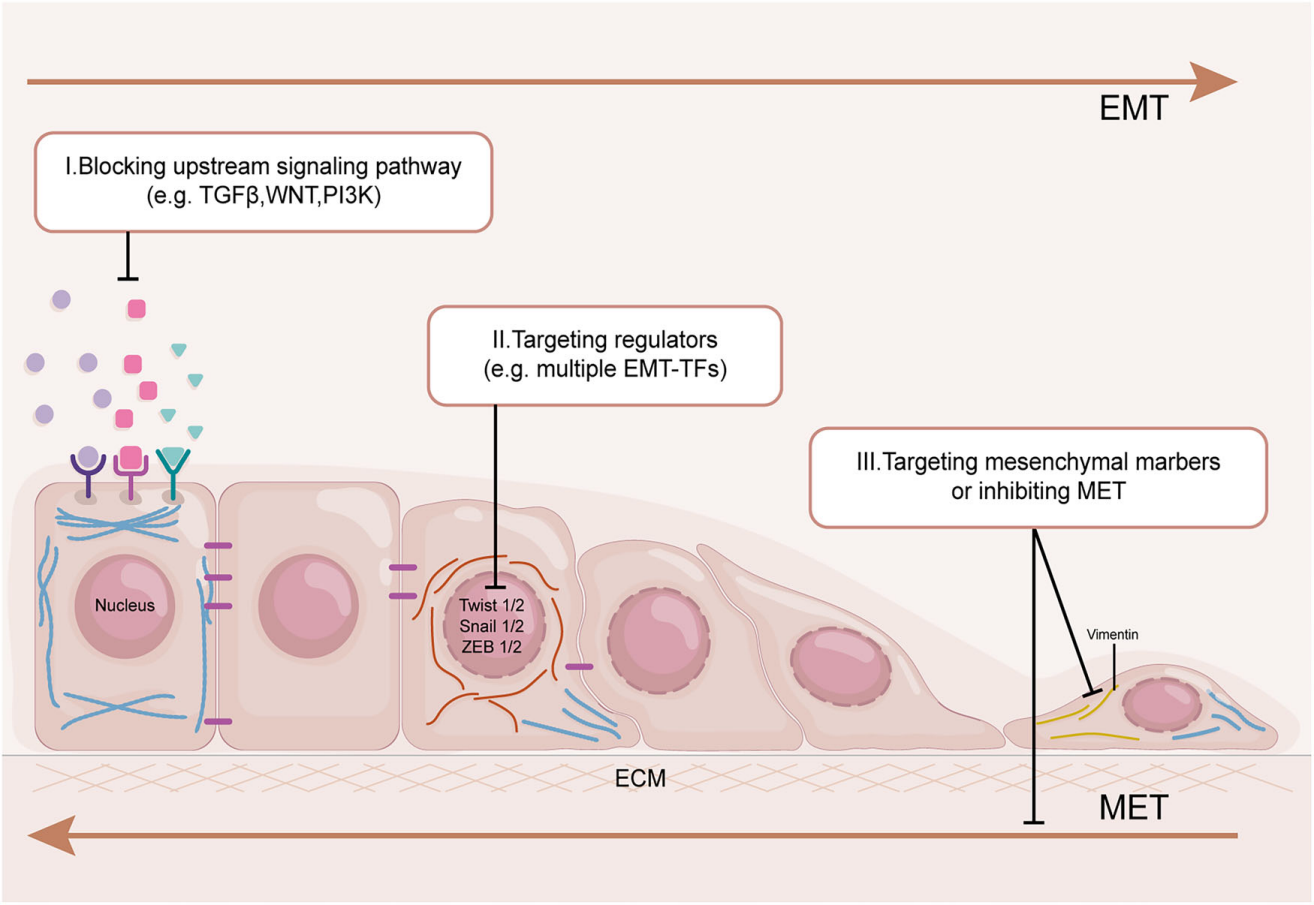
Therapeutic strategies for targeting EMT. There are three main strategies for targeted EMT treatment: I) inhibiting tumorigenesis by blocking upstream signaling pathways, II) targeting the molecular drivers of EMT, and III) targeting mesenchymal markers or inhibiting the MET process.

Several strategies for targeted EMT therapy in CRC have been explored (Fig. 5) (Table 1). One primary approach involves indirectly inhibiting EMT by targeting upstream signaling pathways. Experimental and clinical trials have evaluated small-molecule inhibitors, monoclonal antibodies, and multi-target inhibitors targeting pathways including TGF-β, Wnt, VEGFR, and EGFR, as well as key molecules like AKT, KRAS, and COX-2, to suppress EMT in CRC [137–145]. Another strategy focuses on targeting the molecular drivers of EMT. While direct pharmacological inhibition of EMT-TFs is challenging due to their “undruggable” nature as transcription factors, various approaches are being developed. These include disrupting transcription factor-cofactor/protein/DNA interactions and modulating EMT-TF levels via ubiquitylation or emerging techniques like targeting disordered regions, often aiming to prevent resistance and enhance therapeutic efficacy [146]. Targeting EMT effectors to modulate cell adhesion and cytoskeletal dynamics is also a promising avenue. This involves strategies to restore epithelial cell adhesion, such as methotrexate inducing E-cadherin expression, or inhibiting mesenchymal cell motility, for instance, by overexpressing miRNA-17-5p to decrease vimentin [64, 147]. Reintroducing the MET program to reverse EMT is another approach; cyanidin-3-O-glucoside has shown potential in reversing EMT and restoring epithelial phenotypes in oxaliplatin-resistant CRC cells [148]. However, promoting MET could inadvertently enhance the metastatic potential of already disseminated cells, emphasizing the need for precise treatment windows [149]. Finally, modulating EMT status through alterations in metabolism (e.g., shikonin-loaded nanoparticles inhibiting PKM2 and glycolysis) and the immune microenvironment offers novel targeted delivery and combination strategies for CRC treatment [122].

**Table 1:** Inhibitors of EMT in clinical phase trials.

Drug	Target	Phase	Trial identifier
Blocking upstream signaling pathway			
Galunisertib	TGF-βRI	2	NCT02688712
Vactosertib	TGF-βRI	1 and 2	NCT03724851
SHR-1701	TGF-βRII and PD-L1	2 and 3	NCT04856787
NIS793	Anti-TGF-β antibody	1	NCT02947165
Tegavivint	β-catenin inhibitor	1 and 2	NCT04851119
Regorafenib	RTK inhibitor	Approved	Inapplicable
Cabozantinib	RTK inhibitor	2	NCT03539822
Ipatasertib	AKT inhibitor	2	NCT02465060
sotorasib	KRAS G12C inhibitor	3	NCT06252649
Panitumumab	Anti-EGFR antibody	Approved	Inapplicable
Celecoxib	COX-2 inhibitor	3	NCT01150045
Targeted EMT drivers			
Metformin	Snail and Twist inhibitor	3	NCT05921942
Vorinostat	HDAC1/2/3/6	2	NCT02316340
INCB-59872	LSD1	1 and 2	NCT02959437
Targeting EMT effector molecules			
Methotrexate	Restore E-cadherin expression	Preclinical	Inapplicable
Other strategies			
Cyanidin-3-O-glucoside	Reverse EMT	Preclinical	Inapplicable
SHK@HA-MPDA	PKM2 inhibitor	Preclinical	Inapplicable

## Discussion

Our understanding reveals that cancer cells often adopt partial or hybrid E/M states, conferring enhanced metastatic potential and resilience by combining advantageous epithelial and mesenchymal traits. These intermediate states significantly contribute to intratumoral heterogeneity and adaptive capabilities, posing challenges for mechanistic understanding and therapy. The regulatory networks governing EMT are highly intricate, involving extensive crosstalk between signaling pathways, transcription factors, and epigenetic modifiers, meaning that targeting single components may be insufficient due to compensatory mechanisms [5, 19].

While evidence supports EMT’s role in the invasive cascade and CTC dynamics in CRC, nuances persist regarding the necessity of a complete EMT versus partial states or collective cell migration in all metastatic scenarios [5, 150]. Furthermore, the intimate link between EMT and CSC-like characteristics is critical, as EMT-TFs often co-regulate stemness pathways, endowing cells with tumor-initiating capabilities essential for metastatic colonization and relapse [9]. This EMT-stemness axis represents a significant therapeutic target for eradicating cells responsible for long-term tumor propagation and recurrence. Furthermore, the dynamic interplay between EMT and TME adds complexity. The TME regulates EMT through various signaling molecules, and EMT-activated cells reciprocally remodel the TME, often fostering an immunosuppressive niche that aids immune evasion and can hinder the efficacy of immunotherapies [8]. Concurrently, metabolic reprogramming, including shifts towards glycolysis (the Warburg effect) and altered fatty acid or glutamine metabolism, fuels the energetic demands of invasive cells undergoing EMT [10]. These interconnections demonstrate that EMT is not an isolated cellular program but is deeply integrated with the broader tumor ecosystem, influencing and being influenced by its surroundings.

Developing reliable EMT-associated biomarkers for CRC prognosis and therapeutic guidance is complicated by EMT’s dynamic and reversible nature. While multi-marker panels and CTC analysis show promise for real-time monitoring [11], the need for sequential assessment to capture these fluctuating states is apparent. A formidable challenge remains EMT’s profound contribution to broad therapeutic resistance. Despite compelling preclinical data, translating EMT-targeted therapies into effective clinical treatments for CRC remains difficult, partly due to the “undruggable” nature of many core EMT-TFs and the potential for off-target effects or inadvertently promoting metastatic colonization by inducing MET in disseminated cells.

## Conclusion

EMT is a critical orchestrator of CRC aggressiveness, driving invasion, metastasis, heterogeneity, and therapy resistance through complex molecular interactions. While its plasticity presents therapeutic challenges, a deeper understanding unveils novel vulnerabilities. The future of targeting EMT in CRC necessitates a profoundly dynamic and adaptive therapeutic approach, moving decisively beyond static, “one-size-fits-all” strategies. Critical advancements will hinge on identifying robust, dynamic biomarkers, potentially through non-invasive liquid biopsies, to accurately reflect EMT status, predict prognosis, and precisely guide therapeutic selection. Developing more specific and less toxic inhibitors, alongside highly personalized and adaptive medicine approaches, will be crucial given the inherent tumor plasticity and heterogeneity. Rational combination therapies, integrating EMT inhibitors with conventional chemotherapy, radiotherapy, other targeted agents, or immunotherapy, hold significant promise. Furthermore, a deeper understanding of partial EMT states and strategically modulating the TME and metabolism are essential to bridge preclinical insights with safe and efficacious clinical interventions, ultimately improving patient outcomes.

## Data Availability

The data for this study are available from the corresponding author on reasonable request.
